# Performance of self-cured versus light-cured universal adhesive in patients with non-carious cervical lesions: 18-month randomized clinical trial

**DOI:** 10.1038/s41405-024-00204-9

**Published:** 2024-03-19

**Authors:** Aya Gamal Ashour, Rawda Hesham Abd ElAziz, Asmaa Ali Yassen

**Affiliations:** 1 Conservative Dentistry Department, Faculty of Dentistry, Cairo University, Cairo, Egypt; 2 Egyptian Ministry of health, Cairo, Egypt; 3 Faculty of Dentistry, Cairo University, Cairo, Egypt; 4 Conservative and Esthetic Dentistry, Faculty of Dentistry, Cairo University, Cairo, Egypt

**Keywords:** Bonded restorations, Composite resin

## Abstract

**Objective:**

To evaluate the clinical performance of self versus light-cured universal adhesive in non-carious cervical lesions (NCCLs) after 18 months.

**Materials and methods:**

Sixty-eight NCCLs in 28 patients were divided into two equal groups; G1: self-cured universal adhesive (Palfique,Tokuyama,Japan) and G2: light-cured universal adhesive (Single Bond Universal,3 M ESPE,USA). Nanohybrid resin composite (Z350XT, 3 M ESPE, USA) was used as a final restoration. Evaluation for fracture, loss of retention, marginal adaptation and discoloration were done after 1 week, 6,12, and 18 months using FDI criteria. Postoperative sensitivity was assessed after 1 week. Chi-Square and Mann-Whitney tests with statistical significance at (*P* ≤ 0.05) were used for intergroup comparison,while the intragroup one was performed using the Cochran’s Q and Friedman’s tests. Survival rate was analyzed using Kaplan-meier and Log-rank test.

**Results:**

Both groups exhibited fracture and retention loss, however, there was statistically significant difference favoring the control group at 6 months (*p* = 0.0114,0.0016). For secondary outcomes, marginal adaptation and discoloration revealed no significant differences. For postoperative sensitivity, there was a significant difference favoring the control group (*p* = 0.0007, 0.0011). Palfique had 1.5 Relative-Risk (RR) after 6 months (95% CI 0.5659–4.2617; *P* = 0.3928) and 20% less risk of failure after 18 months (RR 0.8) (95% CI 0.4618–1.3858; *P* = 0.4260). Tested adhesives showed equal survival rate (*P* = 0.5685).

**Conclusions:**

Both adhesives revealed similar clinical performance in restoring the NCCLs after 18 m, however, the early failure was more frequent in the self-cured universal adhesive.

## Introduction

Non carious cervical lesions (NCCLs) are lesions that affect the hard tooth structure without any bacterial pathogenesis. They have different causative factors and consequently, they are classified into chemical, mechanical or chemo-mechanical lesion. Their prevalence (NCCLs) was about 46% in adult population and currently raised to 53% in adults above 30 years worldwide [1]. However, there is a lack of knowledge about the prevalence of these lesions among the Egyptian population. Retention loss of restorations placed in NCCLs continues to be a major concern for clinicians, since it can be affected by many factors that include the difficulty of adhesion between tooth structure and the restorative materials, the lesion extension, the degree of dentin sclerosis, and the adequate establishment of the hybrid layer. The direct and indirect forces are other factors that could have a negative impact on the durability of the NCCLs restorations. They include the masticatory forces especially the micro-shear force and the forces of parafunctional habits if exist. Furthermore, the acidic challenge that could affect the bondability of the restorative material in the erosive lesions with chemical etiology. So, there is a huge variety in degree of retention loss ranges from 0 to 50% [2]. This makes these types of lesions the most challenging condition for testing tooth adhesion in the clinical trials.

The idea of dental adhesion was developed in 1949, by Dr. Hagger a Swiss chemist who started using adhesives as a dentinal seal underneath restoration. These adhesives were classified according to the complexity of technique used into different generations which refer to the order by which they were developed. In the early 1990s, a revolution in the adhesive protocol occurred by introducing three-step etch and rinse adhesive system that was later simplified to two-step etch and rinse and two-step self-etch 2 adhesive systems. In the late 1990s another modification in the form of All-in-one adhesive was done to facilitate the bonding procedures through reducing the multiple steps system with more simplified application procedures to reduce the technique sensitivity, application time and increase patient satisfaction [3]. Multi-mode or universal adhesives have been introduced in the market following the previously mentioned types of adhesives, they can be used with all adhesion strategies after deciding the most suitable strategy for each type of tooth substrate whether it is enamel or dentin [4]. The rationale behind their introduction was to decrease steps and technique sensitivity by providing a single product suitable for all different strategies and offering more privilege due to their chemical bonding to tooth structure with its unique composition [5]. Majority of these adhesives were light cured to take the privilege of the command setting and maturity of the hybrid layer at the time of composite application. However, light cured adhesives face a great challenge in deep cavities and in areas inaccessible to light which dictate double or triple the curing time making the application technique longer and sensitive.

Further attempts were done by the adhesive industry to make the procedures less technique sensitive and more successful. One of these attempts was to eliminate the light curing step, this will not only counteract the problem of incompatibility with dual cured resin cement while cementing the indirect tooth-colored restorations but also will help in bonding of direct resin composite restorations in cases facing difficulty for light to reach as in deep inaccessible areas. Such recommendations were adopted by the manufacturer, Tokuyama, Japan [6]. Currently all the available literature that assessed the performance of this self-cured adhesive was in vitro studies, that is why this study was conducted [7, 8]. It was aimed to analyze the clinical performance of this adhesive in comparison to the light cured universal one in restoring cervical NCCLs. The null hypothesis tested stated that there is a similar clinical performance of both adhesives after 18 months follow up.

## Methods

The current clinical study was performed using the protocol described by the Consolidated Standards of Reporting Trials (CONSORT) and conducted according to the ethical principles outlined in the World Medical Association Declaration of Helsinki. This two-parallel armed randomized control clinical trial was held in the outpatient clinic of the Conservative Dentistry Department, Faculty of Dentistry, Cairo University, after registration at www.clinicaltrials.gov by ID number NCT04572386 and ethical approval number 11.9.20, from October 2020 to April 2023 with equal allocation ratio of 1:1. As the broadest applicability of findings comes from randomized controlled trials, it was recommended to include the sample per patient (two-parallel armed) not per tooth to increase patient variations and consequently increases the applicability [9, 10]. Simple randomization was conducted by computerized sequence generation by someone who is not involved in the research. Participants, statisticians, and the outcome assessors were blinded. Details of the materials used are presented in Table 1.

**Table 1:** List of materials’ names, descriptions/composition, serial numbers, lot numbers, manufacturers or supplier’s names and websites.

Material name	Description/ Composition	Serial no./LOT no.	Manufacturer or supplier name and website
Etchant: Acido gel	37% Phosphoric acid etchant, an organic Thickening agent, Stain, water, Chlorhexidine Digluconate.	8032240001	Maquira Industria-Portugal https://maquira.com.br/produto/acido-gel-fluoridrico-10/
Palfique Universal Bond	Self-cured universal adhesive comes in 2 bottles. A: Phosphoric acid monomer, New 3D-SR monomer, MTU-6, HEMA, BIS-GMA, TEGDMA, Acetone B: γ-MPTES, Borate, Peroxide, Acetone, Isopropyl alcohol, Water	4548190152049 LOT:099E30	Tokuyama, JAPAN https://tokuyama-dental.com/
Single Bond Universal bond (SBU)	The light-cured universal adhesive is supplied in one bottle that contains 10- MDP Phosphate Monomer, Dimethacrylate resins, HEMA, Vitrebond Copolymer, Filler, Ethanol, Water, initiators, and Silane.	LOT:10226 A	3 M ESPE, USA https://www.3m.com
Filtek Z350 XT	Nano-hybrid resin resin composite contains bis-GMA, UDMA, TEGDMA, and BIS-EMA resin. To moderate the shrinkage, PEGDMA has been substituted for TEGDMA resin. The fillers are a combination of non-agglomerated/non-aggregated 20 nm silica filler, non-agglomerated /non-aggregated 4–11 nm zirconia filler (comprised of 20 nm silica and 4 to 11 nm zirconia particles) (Dentin shades)	70-2010-5989-9 LOT: NC93014	3 M ESPE, USA https://www.3m.com

### Sample size calculation

The sample size was calculated based on a previous study by Perdigao et al. in 2014 in which percentage of successful restorations of NCCLs performed by universal adhesive preceded by selective enamel etching can be observed in 98% of cases [11]. By implementing a two tailed Z test for difference between two independent proportions with an alpha level of 5% and a power of 80%. The minimum sample size needed was 30 teeth per group to detect a difference of 25%. Sample size was increased by 10% to compensate for possible dropouts to reach 34 teeth per group. Sample size was performed using G*Power version 3.1.9.2 for windows.

### Patients’ enrollment

Twenty-eight patients with 68 NCCLs who matched the eligibility criteria were enrolled after signing an informed consent for ethical and legal issues. Patients’ demographic distribution is presented in Table 2. NCCLs were further categorized according to the Smith and Knight wear index depending on teeth contour and depth of lesion and Sclerosis index which categorized the degree of sclerosis according to the lesion color, recorded after the cavity preparation to control the lesion diminution limits as shown in Table 3 [12, 13]. After 18 months 56 restored cavities were assessed with 81.2% retention rate.

**Table 2:** Patient’s demographic data.

		Palfique		SBU		*P* value
		*n*	%	*n*	%	
Gender	Male	9	64%	11	79%	*P* = 0.4113
	Female	5	36%	3	21%	
Arch	Upper	16	47%	22	65%	*P* = 0.7979
	Lower	18	53%	12	35%	
Side	Left	19	56%	15	44%	*P* = 0.3356
	Right	15	44%	19	56%	
Teeth	Anterior	14	41%	21	62%	*P* = 0.0918
	Posterior	20	59%	13	38%	

*P*: probability level is significant at *P* ≤ 0.05.

**Table 3:** Sclerosis and wear indices distribution.

Score		Palfique	SBU
Sclerosis index distribution			
1	*N*	20	28
No sclerosis	*%*	59%	82%
2	*N*	8	6
<50%	*%*	24%	18%
3	*N*	6	0
>50%	*%*	18%	0%
Wear index distribution			
1	*N*	1	0
Minimal	*%*	3%	0%
2	*N*	17	23
Less than 1 mm depth	*%*	50%	68%
3	*N*	16	11
1–2 mm depth	%	47%	32%

After controlling or removing etiological factors, adult patients aged 25–55 with NCCLs and low to moderate caries risk were included. Patients who could not return for recall appointments or had abnormal oral or mental conditions, untreated extra occlusal stresses, TMJ problems, systemic conditions, or having less than 20 teeth under occlusion were excluded. Regarding teeth inclusion criteria, vital teeth with NCCLs, sclerosis index and wear Index from 1–3, and having normal or minimum mobility (grade 1) were included. On the contrary, teeth with cervical caries, periodontal disease that may affect the prognosis of the restoration or the tooth itself, severe hypersensitivity, pulpal involvement or necrosis, and intense subgingival lesion that prevents rubber-dam isolation and violates the biological width were excluded. Thorough information regarding patient dental and medical history with any predisposing factors were collected using a customized diagnostic chart, including ADA caries risk assessment. Extra and intra-oral examinations were performed to identify the etiological factor to facilitate removing or reducing the etiological factor preoperatively, (Table 4).

**Table 4:** Instructions and procedural steps to control any predisposing factors.

Lesion	Instructions to reduce/ treat the cause
Abfraction	Behavioral modification
	1 Instruct the bruxer to stop coffee at night.
	2 Limit the physical or mental activity before going to bed.
	3 Hot packs/ massaging on muscles
	Occlusal / restoration adjustment
	Elimination of high spots and minor occlusion adjustment
	Habit breaking appliance (mouthguard)
	Mouth guards were fabricated to patients with para functional habit. They were instructed to wear mouthguards during sleep hours or stress hours. The habit was noticed during awake time, and they were given maintenance tips for the appliance as:
	• Rinsing with cold water before and after use
	• Hot water should not be used to avoid distortion.
	• A small toothbrush with toothpaste under running water, could be used to clean mouthguards to maintain freshness without exerting pressure to avoid scratching.
	Socking in mouthwashes contain chlorhexidine should be done with caution to avoid staining
Erosion	Habit control measure
	1 Reduce the consumption of lemon / of carbonated soft drinks.
	2 Avoid swishing /holding acidic drink in mouth.
	3 Use a straw when drinking acidic drinks to decrease erosion. risk
	4 Drink plenty of water after any acidic drink consumption
	5 Avoid teeth brushing after consumption of acidic drinks.
	6 Use sodium bicarbonate solution to buffer the acid effect.
	7 Use of sugar free chewing gum (Arabic gum) to stimulate the saliva and buffer the effect of acid.
	8 No mouth guard or any type of appliance was done to those patients to prevent any aggravation of the lesion.
	9 Although fluoride varnish/ reminerlizing agent will help in controlling the lesion progression, it was avoided in this study to prevent any confounders (affect the restoration)

#### Assessor calibration

Clinical assessment of the restorations by FDI criteria was done by two expert blinded assessors. Furthermore, calibration sessions for the two assessors (AS and RH), one month before the assessment appointment were conducted till a high degree of agreement was achieved between them. Such calibration was calculated on 10 patients not included in the trial. In case of disagreement extended in depth discussion was done till reaching consensus. Intra and inter assessor agreement was tested by Cohen’s Kappa test after the calibration sessions and it was excellent (97 and 99% respectively).

#### Cavity preparation and restorative procedures

**Fig. 1:**
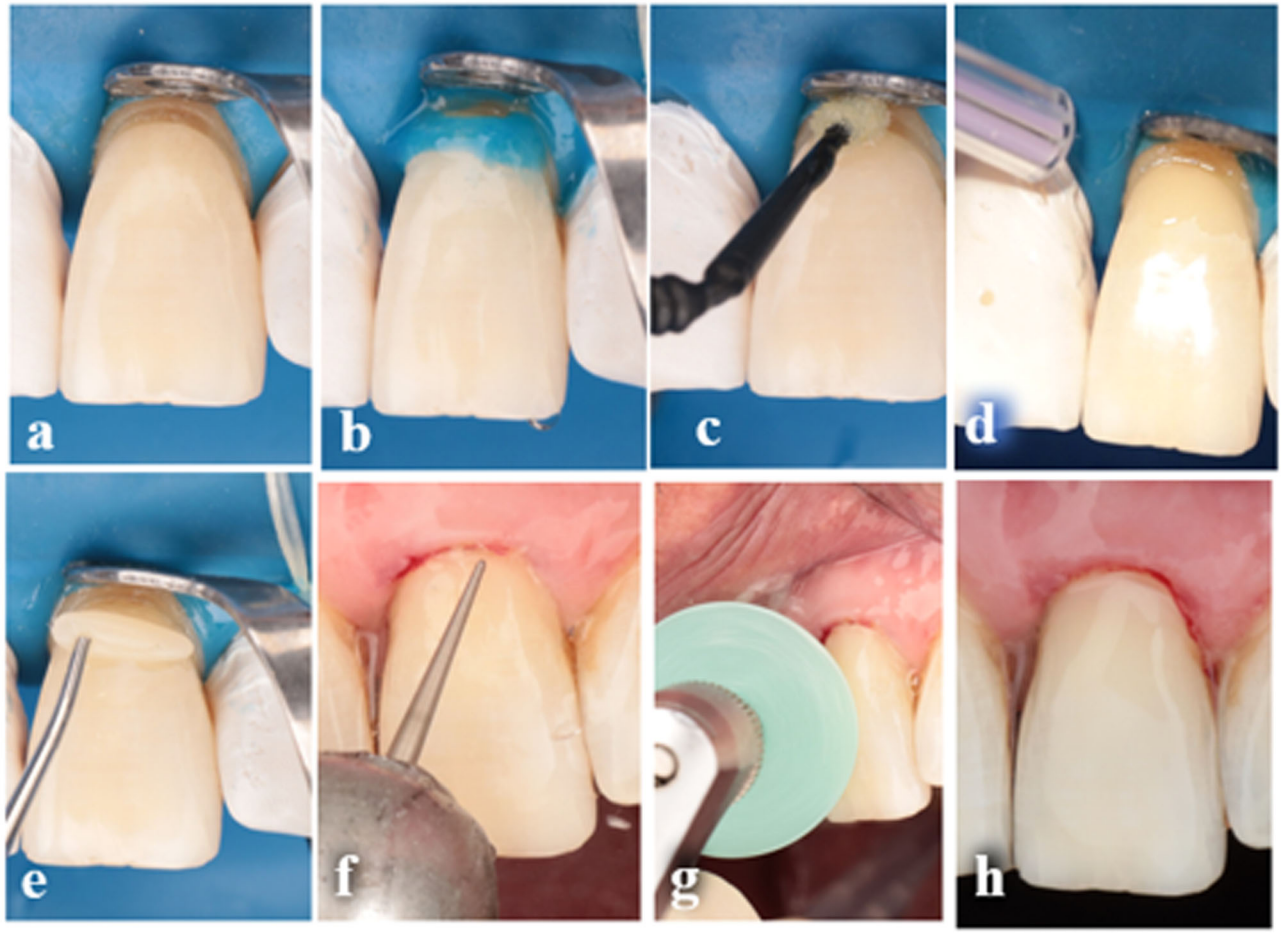
Application steps of self-cured adhesive. **a** Cavity after Isolation, **b** selective enamel etching, **c** application of adhesive, **d** air drying till no adhesive movement observed, **e** composite application (roll as sausage shape to facilitate shape contouring in 1 or 2 increments according to cavity size), **f** finishing of restoration using yellow coded fine tapered stone, and **g** finishing of restoration using finishing disk of TOR-VM, **h** final restoration.

Following the prophylaxis session, teeth were anesthetized using Artpharmadent 1:100,000 (Articaine /epinephrine) to avoid any pain during isolation and cavity preparation and to minimize the outward flow of the dentinal fluids during bonding procedures. Incisal/occlusal enamel beveling (1–1.5 mm) was performed using a high-speed red-coded tapered stone (MANI, Japan) in 45° angulation under water coolant [14]. Stones were discarded after a maximum of 5 teeth. Under rubber dam isolation, selective enamel etching for 20 s was conducted. Palfique Universal Bond (Tokuyama, Japan), which is a two-bottle self-cured universal dental adhesive system, was used as an intervention. Adhesive bottles were shaken thoroughly before use, and one drop from each bottle was dispensed at equal proportion and then mixed by a micro-brush till a bluish color was observed. Then, two coats were applied to the previously prepared cavity by rubbing action for 10 s to ensure proper infiltration of the bond. As instructed by the manufacturer, there is no need for waiting, only gentle air thinning until no adhesive movement is seen, then directly proceed with nanohybrid resin composite application (Filtek Z350 XT), (Fig. 1).

**Fig. 2:**
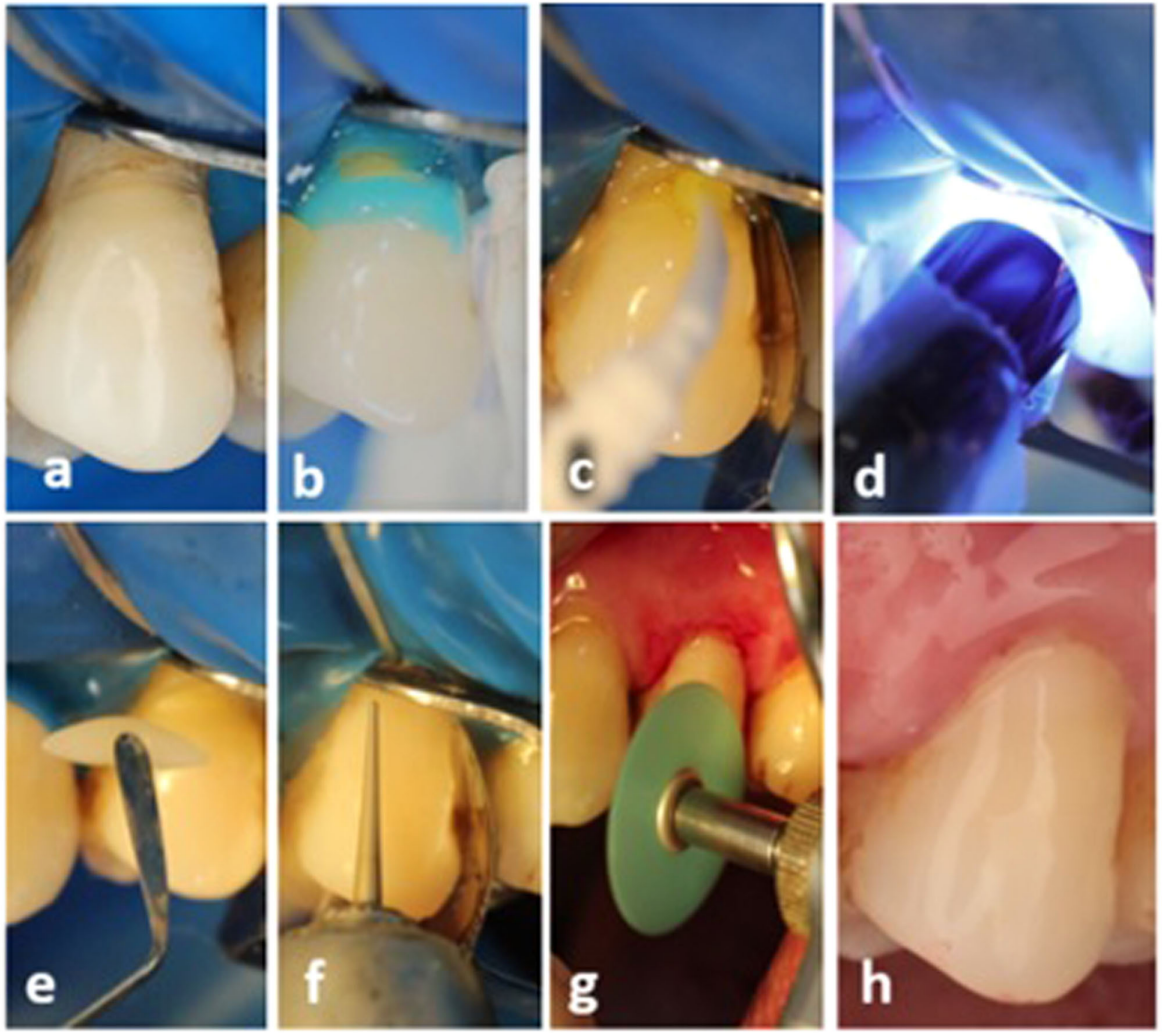
Application steps of light-cured adhesive. **a** Cavity after isolation, **b** selective enamel etching, **c** application of adhesive, **d** light curing for 20 s, **e** composite application (roll in sausage shape to facilitate shape contouring in 1 or 2 increments according to cavity size), **f** finishing of restoration using yellow coded fine tapered stone, and **g** finishing and polishing of restoration using finishing disk of TOR-VM, **h** final restoration.

For the comparator group, light cured Single bond universal adhesive SBU (3 M ESPE, USA) was used. One drop was directly dispensed on the micro-brush, then applied on the prepared cavity using rubbing action. Again, two coats were applied, left undisturbed for 10 s each, gentle air thinned with water/air tip for 10 s, and light cured after the second layer for 20 s at zero distance from cavity margins and in a perpendicular direction using calibrated LED light curing unit (LED F, Woodpecker, China) with 1600 mW/cm^2^, as shown in Fig. 2 [15].

Suitable dentin shade from the nanohybrid resin composite (Z350 XT, 3 M oral care, USA) was used for both intervention and control groups to ensure standardization of the restoration. This type was selected as it has shown superiority in surface smoothing, color stability, and esthetics [16, 17]. Composite was added in increments; each increment was shaped as a sausage to facilitate placement, adaptation, and reaching proper contour using an Elephant composite applicator (EL2). Each increment was light-cured for 20 s using the same curing unit from zero distance.

For finishing, high-speed, yellow-coded tapered stone with a rounded end (MANI, Japan) operated at 10,000 rpm under water coolant in one direction was used, followed by sequential TOR VM disks and EVE polishing tips, giving a finalized smooth surface.

### Outcomes

**Fig. 3:**
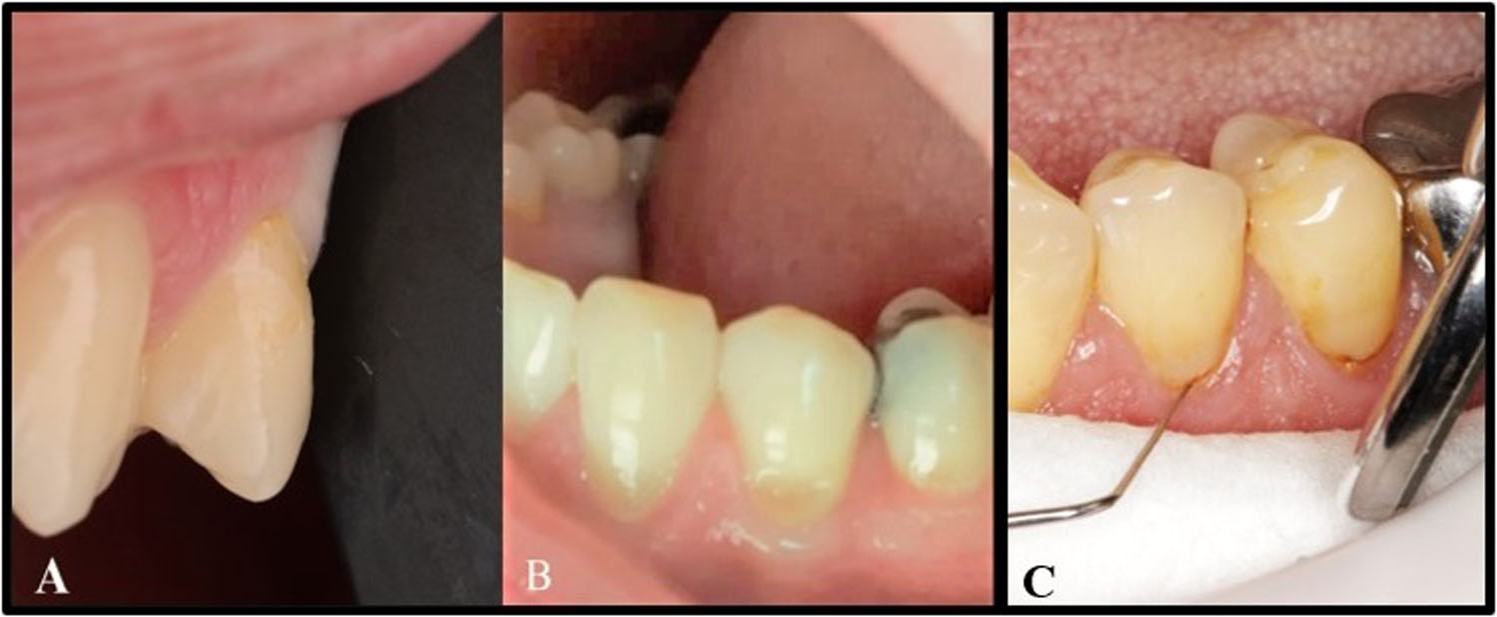
Functional criteria (fracture and retention). **A** Fractured restoration in upper first premolar, **B** lost restoration in lower first premolar, **C** esthetics criteria (marginal discoloration) in lower first molar.

**Fig. 4:**
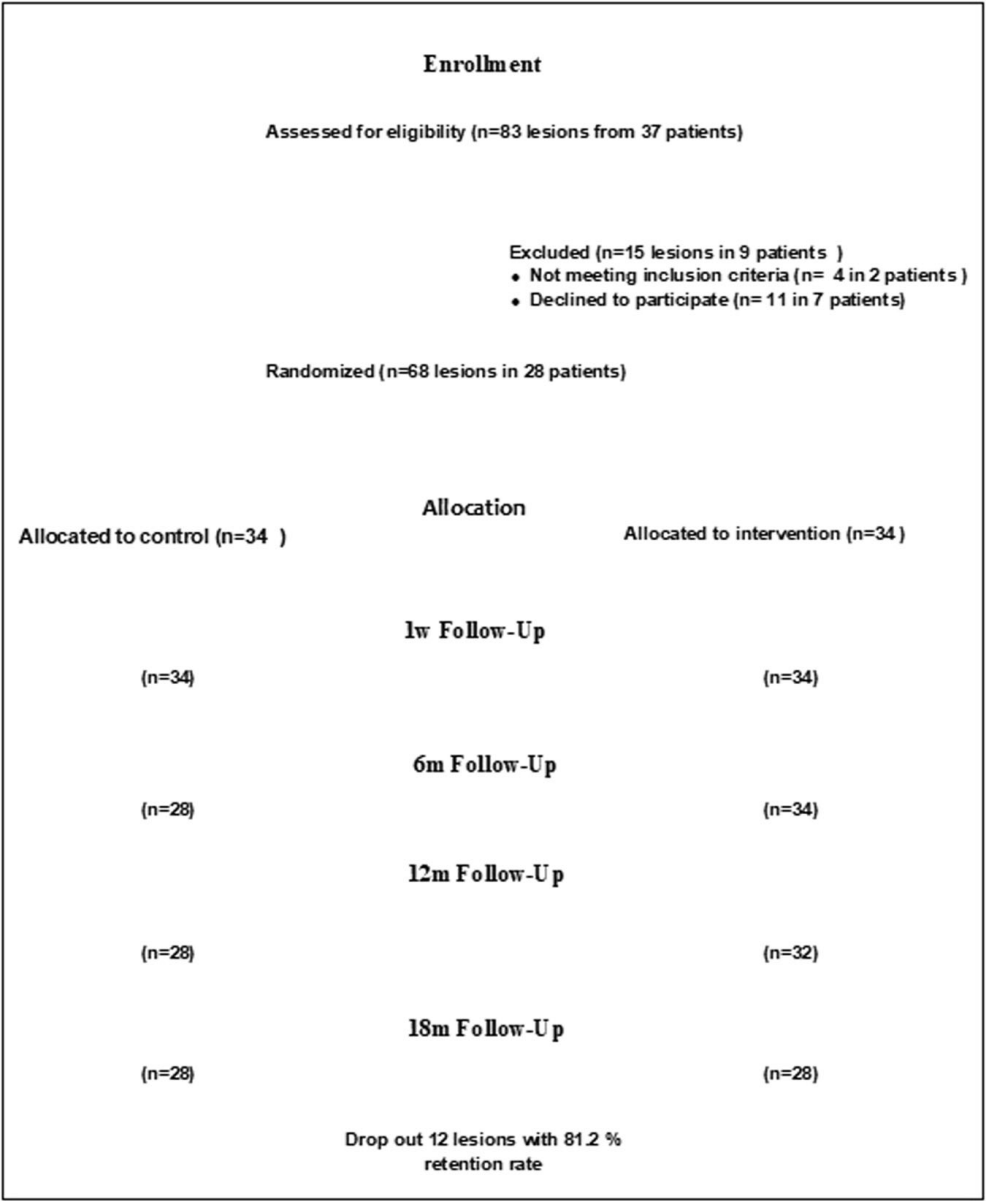
Consort flow chart. Flow chart for the assessed restorations during the different follow-up periods.

The restorations were evaluated following FDI criteria [18, 19]. Both primary (Fracture and loss of retention) and secondary outcomes (Marginal adaptation and discoloration) measurements were recorded after one week, 6, 12, and 18 months. First, a visual evaluation was performed to ensure no retention loss, and then tactile evaluation using an FDI probe (150 and 250 microns) was conducted to check the rocking, cracking, or ditching according to the FDI guidelines. The probe was moved gently at a right angle from tooth to restoration and vice versa, Fig. 3 [14]. Post-operative sensitivity was evaluated after a one-week follow-up using the visual analog scale. A triple air syringe was used at the cavity margin for 5 s to trigger the stimulus and facilitate the identification of pain expression. The follow up of the patient is demonstrated in consort flowchart, Fig. 4 The lost patients during follow up were treated in intention to treat strategy.

### Statistical analysis

Data was analyzed using Medcalc software, version 19 for windows (MedCalc Software Ltd, Ostend, Belgium). Ordinal data was described as frequency and percentage, intergroup comparisons between interventions was performed using Chi-Square and Mann–Whitney tests with statistical significance level set at (*P* ≤ 0.05), intragroup comparison within each intervention was performed using the Cochran’s Q and Friedman’s tests with statistical significance level set at (*P* ≤ 0.0083) after Bonferroni correction. Relative risk was used to assess the clinical significance. Survival rate was analyzed using Kaplan-meier and Log-rank test. The confidence limit was set at 95% with 80% power and all tests were two tailed.

## Results

In the current study intergroup comparison between both adhesives using Mann-whitney and Chi-square tests revealed no statistically significant differences for all tested outcomes at all follow-up periods (*p* > 0.05), except for postoperative sensitivity after 1 week (*p* = 0.0007) and fracture and retention at 6 months (*p* = 0.0114) where there was significant difference favoring Single bond universal (*P* < 0.05). Intragroup comparison within each adhesive using Friedman’s and Cochran’s Q tests revealed statistically significant differences between follow-up periods (*p* < 0.0083), except for marginal adaptation and discoloration within Palfique adhesive, where there was no statistically significant difference (*p* > 0.0083) using Cochran’s Q test (*p* = 0.194 and 1.0000 respectively) (Table 5).

**Table 5:** Frequency and percentages of all score’s distribution regarding A: Fracture and retention loss, B: marginal adaptation, C: marginal discoloration, and D: post-operative sensitivity in both groups and comparison between them.

Outcome	Follow-up	Palfique Universal								Single Bond Universal								P value (Mann–Whitney)	*P* value (Chi-square)
		*N*	Rank	Success				Failure		*N*	Rank		Success			Failure			
				1	2	3	4		5			1		2	3	4	5		
Fracture and Retention	1 week	34	a	34 (100%)	0 (0%)	0 (0%)	0 (0%)		0 (0%)	34	a	34 (100%)		0 (0%)	0 (0%)	0 (0%)	0 (0%)	*P* = 1.0000	*P* = 1.0000
	6 months	34	b	19 (55.8%)	3 (8.8%)	4 (11.8%)	0 (0%)		8 (23.6%)	28	a	26 (92.9%)		1 (3.6%)	0 (0%)	0 (0%)	1 (3.6%)	*P* = 0.0016*	*P* = 0.0114*
	12 months	32	b	15 (46.9%)	5 (15.6%)	3 (9.4%)	1 (3.1%)		8 (25%)	28	b	15 (53.6%)		4 (14.3%)	2 (7.1%)	5 (17.9%)	2 (7.1%)	*P* = 0.4391	*P* = 0.1752
	18 months	28	b	10 (35.7%)	6 (21.4%)	2 (7.1%)	2 (7.1%)		8 (28.6%)	28	b	15 (42.9%)		3 (8.6%)	1 (2.9%)	4 (11.4%)	5 (14.3%)	*P* = 0.2637	*p* = 0.4492
	P value (*Friedman’s*)		P < 0.00001*								P < 0.00001*								
	P value (*Cochran’sQ*)		P < 0.001*								P < 0.001*								
Marginal adaptation	1 week	34	a	34 (100%)	0 (0%)	0 (0%)	0 (0%)		0 (0%)	34	a	34 (100%)		0 (0%)	0 (0%)	0 (0%)	0 (0%)	*P* = 1.0000	*P* = 1.0000
	6 months	27	b	20 (74.1%)	3 (11.1%)	4 (14.8%)	0 (0%)		0 (0%)	27	ab	24 (88.8%)		3 (11.2%)	0 (0%)	0 (0%)	0 (0%)	*P* = 0.1398	*P* = 0.1128
	12 months	24	b	12 (50%)	8 (33.3%)	3 (12.5%)	1 (4.2%)		0 (0%)	27	b	15 (55.5%)		5 (18.5%)	2 (7.5%)	5 (18.5%)	0 (0%)	*P* = 0.8944	*P* = 0.2923
	18 months	20	b	8 (40%)	7 (35%)	3 (15%)	2 (10%)		0 (0%)	23	b	13 (15.6%)		5 (21.7%)	1 (4.3%)	4 (17.5%)	0 (0%)	*P* = 0.4677	*P* = 0.3923
	P value (*Friedman’s*)		P = 0.00002*								P < 0.00001*								
	P value (*Cochran’sQ*)		P = 0.194								P = 0.004								
Marginal Discoloration	1 week	34	a	34 (100%)	0 (0%)	0 (0%)	0 (0%)		0 (0%)	34	a	34 (100%)		0 (0%)	0 (0%)	0 (0%)	0 (0%)	*P* = 1.0000	*P* = 1.0000
	6 months	27	b	22 (81.5%)	1 (3.7%)	4 (14.8%)	0 (0%)		0 (0%)	27	ab	23 (85.2%)		0 (0%)	0 (0%)	3 (11.1%)	1 (3.7%)	*P* = 0.9288	*P* = 0.0605
	12 months	24	b	16 (66.7%)	2 (8.3%)	6 (25%)	0 (0%)		0 (0%)	27	b	19 (70.4%)		3 (11.1%)	1 (3.7%)	3 (11.1%)	1 (3.7%)	*P* = 0.9282	*P* = 0.0961
	18 months	20	b	11 (55%)	3 (15%)	6 (30%)	0 (0%)		0 (0%)	23	b	13 (56.5%)		0 (0%)	6 (26.1%)	3 (13%)	1 (4.4%)	*P* = 0.5301	*p* = 0.1363
	P value (*Friedman’s*)		P = 0.00046*								P = 0.00392*								
	P value (*Cochran’sQ*)		P = 1.0000								P = 0.007*								
Postoperative Sensitivity	1 week	34	b	24 (70.5%)	10 (29.5%)	0 (0%)	0 (0%)		0 (0%)	34	a	34 (100%)		0 (0%)	0 (0%)	0 (0%)	0 (0%)	*P* = 0.0011*	*P* = 0.0007*

There was 1.5 times more risk for failure in Palfique group when compared to SBU after 6 months, RR 1.5 (CI (0.5659–4.2617) at *p* = 0.3928). On the other hand 20% less risk of failure was detected in Palfique adhesive when compared to SBU after 18 months, RR 0.8 (CI (0.4618–1.3858) at *p* = 0.4260).

**Fig. 5:**
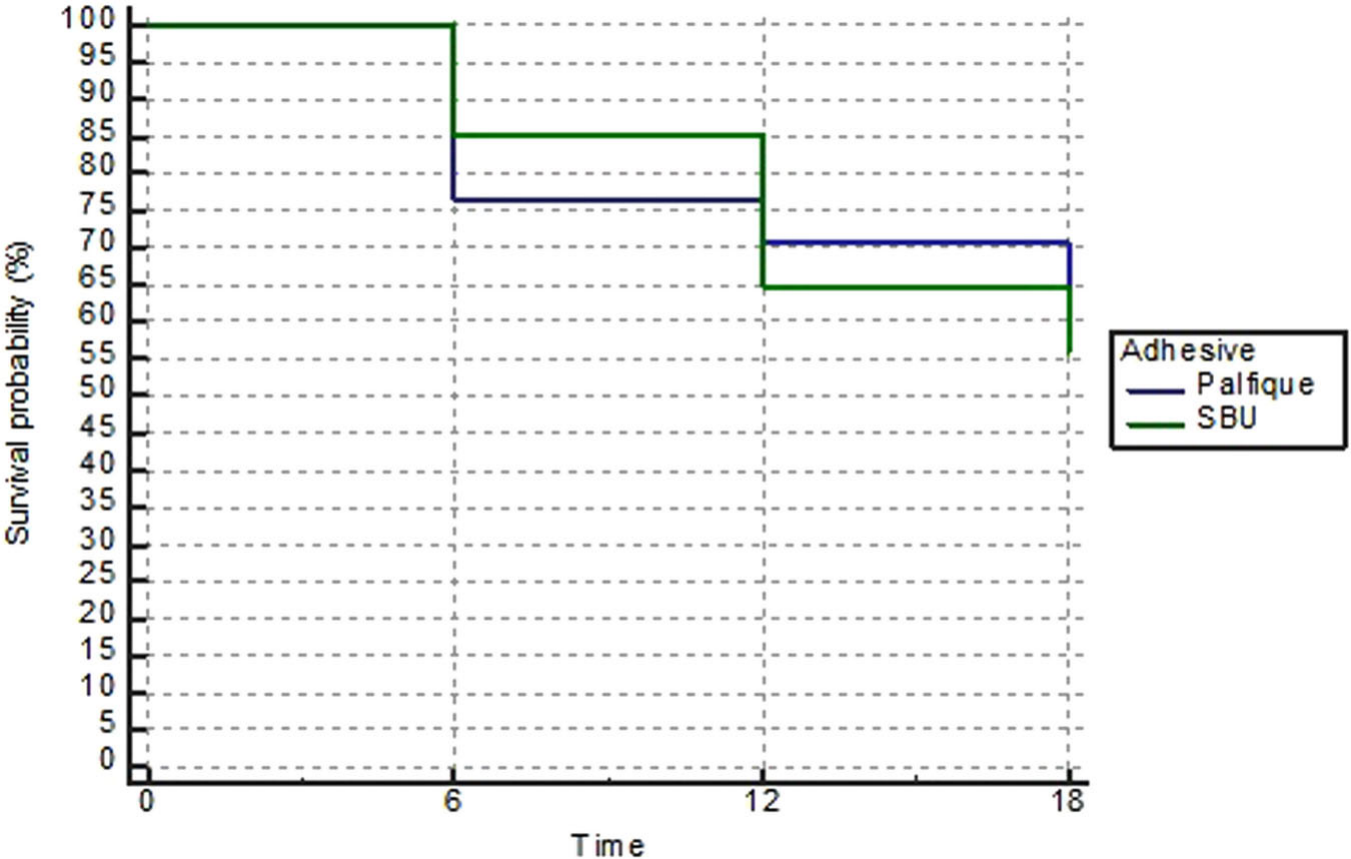
Overall survival. Overall survival of composite restorations for both groups.

Overall survival of composite restorations using both adhesives was assessed after 6 and 18 months. Results revealed that Palfique showed early failure of 8 restorations, while SBU showed early failure of 5 restorations after 6 months with a success rate of 76.5% and 82% respectively. While, there were12 restorations in Palfique group and 15 restorations in SBU group were scored as failed restorations (score 4 and 5) after 18 months using FDI criteria with a success rate of 58% and 46.5% respectively with non statistically significant difference between both adhesives (*p* = 0.5685). (Fig. 5).

## Discussion

Achieving successful and long-lasting bonding to tooth structure is one of the most important goals for any dentist wants to practice minimal invasive dentistry. However, this goal is not easy to be achieved due to several challenges facing the clinicians like the substrates involved and the choice between many available adhesives. Each substrate and adhesive dictate certain protocol for bonding. Unfortunately, multiple published laboratory studies have tested different dental adhesives’ properties but they did not reflect their clinical performance [20]. Therefore, this is the first study to evaluate the clinical performance of self-cured universal adhesive (Palfique,Tokuyama,Japan) in comparison to one of the most commonly used light cured universal adhesive (Single Bond Universal,3 M ESPE,USA). It was claimed that Palfique has low technique sensitivity as stated by the manufacturer in addition, it is supplied in two bottles, which gives it the advantage of being more stable, as curing will only happen after mixing. Another important issue is related to its composition, which contains borate self-etch (BoSe) with 3d-SR technology. Three-dimensional cross-linking occurs by calcium interaction, adhesive 3D-SR monomers, and other monomers that lead to optimal fast polymerization and a strong bonding layer. This process occurs by contact cure, where the phosphoric acid monomer breaks down the borate initiator, turning it into borane molecules, which generate free radicals. Moreover, peroxide accelerates the degradation of the borane compound, which acts as a highly active chemical polymerization initiator. When this bonding layer encounters resin composite, it rapidly progresses through polymerization and becomes cured on its adhesive interface (contact-cure). However, the clinical performance of this adhesive is not fully approved in the literature. On the other hand, the light-cured universal adhesive is characterized by its ability to bond chemically to the tooth structure owing to the presence of 10-MDP functional monomer and polyalkenic acid [21, 22].

Clinical performance of these adhesives was tested in NCCLs as the retention here depends mainly on the bond quality without any mechanical retention support. Moreover, these lesions have sclerotic dentin which is considered the most problematic bondable coronal substrate as mentioned in the introduction. ^20^It is not easily etched like normal dentin because of its ultrastructure, including hypermineralization on the surface with denatured collagen and bacteria invasion with obliteration of the dentinal tubules by crystalline deposits. In the current study, the most common sclerosis scores were 1 and 2, which show weak or no signs of sclerosis, while score 3 was only detected in the intervention group. This could be one of the reasons that explains the increased failure rate regarding the early retention loss in the intervention group. The FDI criteria were also selected to evaluate the restoration because of its detailed and meticulous evaluation parameters. It includes three main parameters considering functional, esthetic, and biological, with 16 subcategories, from 1 to 5, according to detailed, clear guidelines [18] that enable the clinician to easily quantify and evaluate the restoration change in short-term clinical studies. It is worth mentioning that the leading cause of NCCLs restoration failure is the retention loss and fracture [23, 24]. That is why retention loss and fracture were taken as a primary outcome, followed by marginal adaptation and marginal discoloration [25].

Fracture and retention loss results revealed a statistical decrease in FDI score 1 and a significant increase in FDI scores 2, 4, and 5 from 1 week to 18 months, indicating the increase in the rate of restoration deterioration ranging from minor marginal chip fracture to complete restoration loss. Furthermore, there are statistically significant differences between both tested groups at 6 months, favoring the comparator as it showed less fracture and retention loss. This can be attributed to the degree of conversion of the adhesives when using a light cure. Light increases the degree of conversion instantly, while in self-cured, it depends on the conversion rate over a certain time, which will be significantly affected by the intraoral condition when put into function as moisture contamination and force of mastication. This is besides the polymerization shrinkage of the resin composite that might negatively affect the self-cured adhesive which was not fully cured at the restoration time. Furthermore, in the light cured adhesive, the functional monomers with strong and stable chemical affinity to hydroxyapatite improved adhesive performance and gave better retention (10-MDP). Moreover, light-cured adhesive exhibited significantly higher shear bond strength values and the lowest nano leakage, followed by self-cured universal adhesive, which attributed to the variation in failure rate between the two bonds [26]. These results were confirmed by the relative risk and survival rate findings of the current study as the self-cured universal adhesive showed 1.5 more failure risk than the light cured one after 6 months and similar survival rate after the 18 months. On the contrary Xu et al. in 2006 stated that the polymerization of light cured adhesive has got negatively affected by increasing the distance between the curing light and the adhesive layer, however, in this study cavities were not too deep for the light to fully reach the adhesive [27].

With reference to marginal adaptation and marginal discoloration, there was a significant decrease in FDI score 1 with a significant increase the other scores from 1 week to 18 months. The control group showed preservation of marginal properties after 6 months compared to the intervention group but without statistically significant difference. At 12 and 18 months, the remaining restorations gave comparable results; consequently, marginal adaptation should not be included alone without the fracture and retention value. This deterioration can be due to minor fractures at the margin caused by the unpolymerized bond that showed weak tensile and shear bond strength. This explanation can correlate bond strength values obtained in the in vitro studies with the clinical outcomes. Besides, the presence of rough margins due to marginal deterioration which can increase the chance of marginal discoloration by being more retentive [28]. On the other hand, Palfique showed higher post-operative sensitivity at 1 week [29]. Most of the cases that reported post-operative sensitivity showed loss of retention in the intervention group, which can be explained by the incomplete polymerization of the self-cured adhesive, making the material more liable to nano leakage. For these reasons and under the circumstances of the current study the null hypnosis was partially rejected.

Finally, it is worth mentioning that within the limitation of this study, both types of adhesives showed similar clinical performance after 18 months in NCCLs. This finding emphasizes the great influence of the oral environmental factors, starting from the nature of tooth structure and going through the human variations such as masticatory force and dietary habits, on the serviceability of the resin composite restorations in the NCCLs.

## Conclusions

Following the current trial’s circumstances and limitations, it can be concluded that,

- Light-cured adhesive may offer some advantages over self-cured one in term of number of restorations with early failures.
- Similar studies might be needed to insure the reliability of self-cured universal adhesive for direct resin composite restorations under different circumstances.

The current study raised some recommendations for the researchers, which are;

- An extended follow-up period is required to evaluate the long-term survival of each group.
- Studying the influence of the degree of sclerosis on the clinical performance of the adhesives will be of great value.
- Comparing the clinical performance of self-cured adhesive in areas away from or not accessible to light with the light-cured adhesive might be necessary.

## Data Availability

Full data are available whenever requested.
